# Palliative care and healthcare utilization among deceased metastatic lung cancer patients in U.S. hospitals

**DOI:** 10.1186/s12904-022-01026-y

**Published:** 2022-07-27

**Authors:** Jongwha Chang, Kyu-Tae Han, Mar Medina, Sun Jung Kim

**Affiliations:** 1 Department of Healthcare Administration, College of Business, Texas Woman’s University, Denton, TX USA; 2 Division of Cancer Control and Policy, National Cancer Center, Goyang, Republic of Korea; 3 National Hospice Center, National Cancer Center, Goyang, Republic of Korea; 4 School of Pharmacy, University of Texas at El Paso, El Paso, TX USA; 5 Department of Health Administration and Management, College of Medical Science, Soonchunhyang University, Asan, Republic of Korea; 6 Center for Healthcare Management Science, Soonchunhyang University, Asan, Republic of Korea; 7 Department of Software Convergence, Soonchunhyang University, Asan, Republic of Korea

**Keywords:** Lung cancer, Palliative care, NIS sample, Healthcare utilization

## Abstract

**Objective:**

The benefits of palliative care for cancer patients were well developed; however, the characteristics of receiving palliative care and the utilization patterns among lung cancer patients have not been explored using a large-scale representative population-based sample.

**Methods:**

The National Inpatient Sample of the United States was used to identify deceased metastatic lung cancer patients (*n* = 5,068, weighted *n* = 25,121) from 2010 to 2014. We examined the characteristics of receiving palliative care use and the association between palliative care and healthcare utilization, measured by discounted hospital charges and LOS (length of stay). The multivariate survey logistic regression model (to identify predictors for receipts of palliative care) and the survey linear regression model (to measure how palliative care is associated with healthcare utilization) were used.

**Results:**

Among 25,121 patients, 50.1% had palliative care during the study period. Survey logistic results showed that patients with higher household income were more likely to receive palliative care than those in lower-income groups. In addition, during hospitalization, receiving palliative care was associated with11.2% lower LOS and 28.4% lower discounted total charges than the non-receiving group.

**Conclusion:**

Clinical evidence demonstrates the benefits of palliative care as it is associated with efficient end-of-life healthcare utilization. Health policymakers must become aware of the characteristics of receiving the care and the importance of limited healthcare resource allocation as palliative care continues to grow in cancer treatment.

## Background

Lung cancer is one of the leading causes of cancer-related deaths worldwide. Approximately 1.7 million deaths occur annually, accounting for about 20% of all cancer-related deaths [1]. In 2018, estimated new cases of lung cancer were 234,030 (121,680 men and 112,350 for women) and accounting for 14% of new cancers in men and 13% of new cancers in women in the U.S [2]. Lung cancer is also associated with 154,050 deaths (83,550 for men/70,500 for women) in 2018, which accounted for 25% of all cancer fatalities in the U.S. [2].

Palliative care is medical care aimed at improving the quality of life of seriously ill cancer patients and their families through comprehensive assessment and treatment of the physical, psychosocial, and spiritual areas, including relief of pain and symptoms [3]. Previous research has investigated the benefit of palliative care on lung cancer patients and confirmed that palliative care improves patient and caregiver outcomes, and is also associated with less medical interventions near the end of life [4–8]. With that in mind, the American Association of Clinical Oncology suggested that palliative care should be considered for managing advanced cancer patients [9, 10]. Moreover, cancer patients' healthcare costs and utilization were markedly increased, creating a "U" shape at the first stage of diagnosis and end of life. Especially during the last months of life, hospital charges are known to increase rapidly, a trend confirmed by studies in USS Medicare patients [11–13] and lung cancer patients in Korea, where a single health insurance payer system is also utilized [14].

Palliative care is associated with decreased cost, and as the clinical and economic benefits of palliative care for metastatic cancer patients become evident, more patients are seeking the treatment [15, 16]. A study suggested that the share of palliative care use among all deaths varied by country: the United States (52.0%), the United Kingdom (46.6%), Canada (40.8%), Korea (24.3%), and Taiwan (39.0%) [17]. However, data lagged far behind, and lung cancer-specific results were not well documented. The rapid growth in the number of cancer patients highlights the importance of better understanding the characteristics of palliative care utilization. However, evidence on the use of palliative care with lung cancer has not been reviewed well [18], despite lung cancer being one of the cancer types with a poor prognosis, with meager 5-years survival rates. A recent study investigated the utilization of palliative care using Surveillance, Epidemiology, and End Results-Medicare linked database (2001–2013) and suggested that receipt of palliative care varied significantly by sex, race, and region [15]. Although this study sample is limited to older Medicare population (> 65 years old), it addressed important issues of palliative care 'recipients' characteristics among aged lung cancer patients. Since there is a lack of studies on the aspects of palliative care use by a population representative sample [19, 20] and its association with healthcare utilization among deceased metastatic lung cancer patients, it is crucial to examine the issue.

To address these research gaps, the aim of this study is two specific objectives: 1) to investigate the temporal trend of receiving palliative care and its association with patient characteristics, and 2) to examine how palliative care is associated with efficient healthcare utilization among end life of deceased metastatic lung cancer patients using a representative population sample.

## Methods

### Data collection

**Fig. 1:**
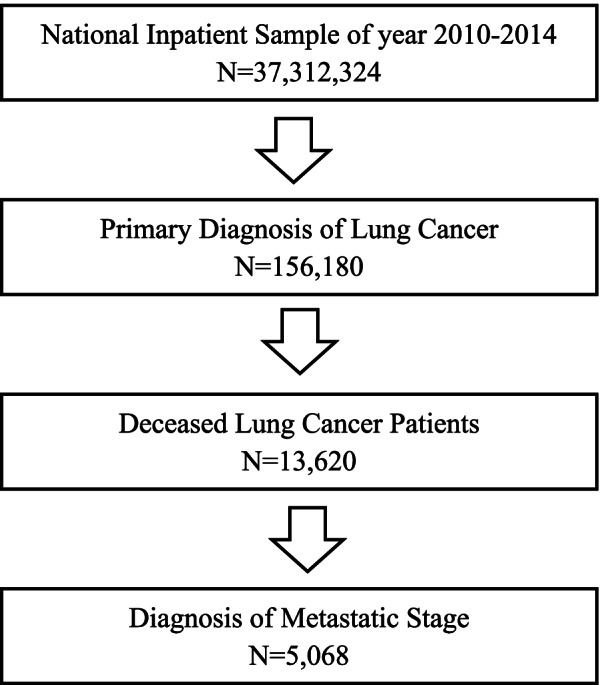
Flow chart of Patient Sample Selection

The 2010–2014 National Inpatient Sample (NIS) data, by Healthcare Cost and Utilization Project.

(HCUP) of Agency for Healthcare Research and Quality (AHRQ), were utilized to obtain patients with metastatic lung cancers. NIS is one of the largest publicly available dataset which include all-payer US hospital inpatients records. Among all 2010–2014 NIS samples (*N* = 37,312,324), as shown in Fig. 1, we first verified a primary diagnosis of lung cancer (total *n* = 156,180) using the International Classification of Diseases, Ninth Revision, Clinical Modification (ICD-9-CM) codes for lung cancer. Then we obtained patients that were deceased (*N* = 13,620) and were at a metastatic stage (*N* = 5,068) (Fig. 1).

### Variables

The main outcomes of this study was to investigate the characteristics of palliative care use by LOS (length of stay) and hospital charges. The main interesting variable was the receipt of palliative care consultation by year. To identify palliative care for hospitalized patients, this study used the ICD-9-CM code of V66.7. Total hospital charges was determined after reflecting for the annual inflation rate using Centers for Medicare and Medicaid Services estimates [21]. Due to the skewness of distribution for hospital charges and length of stay, we conducted the natural log of those variables.

In this study, we adjusted various patient-level confounders. Patient characteristics included age, race, annual median household income, primary payer (Private insurance, Medicare, Medicaid, Self-Pay/No Charge, and Others), number of comorbidities, the severity of illness, and whether the patient received surgery, radiation, or chemotherapy.

### Statistical analysis

In this study, we used sampling weights for statistical analyses to represent deceased metastatic lung cancer patients. First, we examined the characteristics of the final dataset, which include patient characteristics by palliative care use. The patient characteristics were presented as mean or weighted frequency (percentage) with SD (standard deviation). Rao-Scott Chi-Square tests were employed to examine categorical variables for their group differences (whether receiving palliative care).

Using the survey logistic regression analysis, the odds ratios and 95% confidence interval (ORs and 95% CI) for receiving palliative care for deceased metastatic lung cancer patients were calculated. We also investigated how palliative care was associated with discounted hospital charges and length of stay using the multivariate survey linear regression analysis. This study used SAS statistical software (SAS Institute Inc., Cary, NC, USA, Version 9.4) for all statistical analyses. All methods were carried out in accordance with relevant guidelines and regulations in the method section.

## Results

### Patient characteristics

A total of 5,068 deceased metastatic lung cancer were identified in the 2010–2014 NIS data (weighted *n* = 25,121, Table 1). Among them, 2,544 (weighted *n* = 12,588, 50.1%) had palliative care. The general characteristics of patient characteristics are presented in Table 1. The mean LOS and discounted hospital charges were 7.05 days (SD = 7.37 days) and $53,514 (SD = $88,566) for those with palliative care and 8.94 days (SD = 9.15 days) and $81,213 (SD = $114,368) for those without palliative care (Table 1).

**Table 1:** General Characteristics of patient Sample

	**Total**		**Palliative—No**		**Palliative—Yes**		***P***
	***N***	**%**	***N***	**%**	***N***	**%**	
**Unweighted N**	5,068		2,524	49.8%	2,544	50.2%	
**Weighted N** **(National estimates)**	25,121		12,533	49.9%	12,588	50.1%	
**Age Group**							
40	596	2.4%	277	46.5%	319	53.5%	< .0001
40–49	1,683	6.7%	774	46.0%	909	54.0%	
50–59	5,323	21.2%	2,776	52.1%	2,547	47.9%	
60–69	7,900	31.4%	3,944	49.9%	3,956	50.1%	
≥ 70	9,618	38.3%	4,761	49.5%	4,857	50.5%	
**Sex**							
Female	11,843	47.1%	5,733	48.4%	6,111	51.6%	< .0001
Male	13,277	52.9%	6,800	51.2%	6,477	48.8%	
**Race**							
Black	3,482	13.9%	1,961	56.3%	1,520	43.7%	< .0001
Hispanic	1,363	5.4%	727	53.3%	636	46.7%	
Asian or Pacific Islander	801	3.2%	431	53.8%	370	46.2%	
Native American/Other	694	2.8%	366	52.7%	328	47.3%	
White	18,781	74.8%	9,048	48.2%	9,733	51.8%	
**Median household income**							
0-25th percentile	7,340	29.2%	3,972	54.1%	3,367	45.9%	< .0001
26th to 50th percentile	6,151	24.5%	3,133	50.9%	3,018	49.1%	
51st to 75th percentile	5,872	23.4%	2,958	50.4%	2,914	49.6%	
76th to 100th percentile	5,759	22.9%	2,470	42.9%	3,289	57.1%	
**Primary Payer**							
Medicare	12,217	48.6%	6,555	53.7%	5,663	46.3%	< .0001
Medicaid	2,699	10.7%	1,494	55.4%	1,205	44.6%	
Self-Pay/No Charge	1,174	4.7%	532	45.3%	643	54.7%	
Other^a^	1,408	5.6%	379	26.9%	1,029	73.1%	
Private insurance	7,622	30.3%	3,573	46.9%	4,049	53.1%	
**Severity of Illness Subclass**							
APR-DRG 0,1, lowest	3,550	14.1%	1,630	45.9%	1,920	54.1%	< .0001
APR-DRG 2	5,368	21.4%	2,259	42.1%	3,109	57.9%	
APR-DRG 3	9,222	36.7%	4,428	48.0%	4,793	52.0%	
APR-DRG 4, highest	6,981	27.8%	4,215	60.4%	2,766	39.6%	
**Surgery**							
No	24,494	97.5%	12,087	49.3%	12,407	50.7%	< .0001
Yes	627	2.5%	446	71.2%	181	28.8%	
**Radiation**							
No	23,646	94.1%	11,771	49.8%	11,874	50.2%	0.167
Yes	1,475	5.9%	762	51.6%	713	48.4%	
**Chemotherapy**							
No	23,274	92.7%	11,397	49.0%	11,877	51.0%	< .0001
Yes	1,846	7.3%	1,136	61.5%	710	38.5%	
**Number of Comorbidities**^a^	2.76	2.06	2.99	2.12	2.53	1.97	< .0001
**LOS**^a^	8.00	8.36	8.94	9.15	7.05	7.37	< .0001
**Discounted Total Charges**^a,b^	67,364	103,207	81,213	114,368	53,514	88,566	< .0001

^a^Mean/SD

^b^CMS's hospital care (inpatient) inflation rate applied. All discounted at 2010 level

### Patterns of palliative care use

Table 2 shows the temporal trends in palliative care use and healthcare utilization among hospitalized patients with metastatic lung cancer between 2010 and 2014. The rate of receiving palliative care consultations increased from 42.8% to 56.2% during the study period (*p* < 0.001). LOS and discounted total charges were not volatile during the study period.

**Table 2:** Temporal Trend of Palliative care and Healthcare Utilization among deceased metastatic lung cancer

	**Total**	**2010**	**2011**	**2012**	**2013**	**2014**	***P***
**Unweighted N**	5,068	1,075	1,060	897	1,033	1,003	
**Weighted N (National estimates)**	25,121	5,367	5,089	4,485	5,165	5,015	
**Palliative care**							
No	12,533	3,067	2,701	2,065	2,505	2,195	< .0001
Yes	12,588	2,300	2,388	2,420	2,660	2,820	
*% of Yes*	*50.1%*	*42.8%*	*46.9%*	*54.0%*	*51.5%*	*56.2%*	
**LOS**	8.00	8.28	8.33	8.14	7.33	7.90	0.040
**Discounted Total Charges**^a^	67,364	68,684	69,132	67,248	61,496	70,207	0.343

^a^CMS's hospital care (inpatient) inflation rate applied. All discounted at 2010 level

The ORs of receiving palliative care from the survey logistic regression model are shown in Table 3. After controlling for all other variables, age, sex, and race 'didn't play important roles in receiving palliative care. However, the higher household income group was more likely to receive palliative care than the lower household income groups. (Reference group of 76th to 100th percentile, 0-25th percentile OR = 0.639 95% CI = 0.542–0.754, 26th to 50th percentile OR = 0.724 95% CI = 0.613–0.855, 51st to 75th percentile OR = 0.733 95% CI = 0.620–0.866). Additionally, palliative care was significantly less likely to be used in patients receiving surgery (OR = 0.519, 95% CI = 0.341–0.791) or chemotherapy (OR = 0.713, 95% CI = 0.563–0.903) during the same hospitalization.

**Table 3:** Results of Survey Logistic Regression: Odds of Receiving Palliative care among deceased metastatic lung cancer

	**Odds Ratios**	**95% CIs**	
**Age Group**			
40	Reference		
40–49	1.020	0.659	1.579
50–59	0.754	0.508	1.118
60–69	0.889	0.600	1.319
≥ 70	1.000	0.669	1.494
**Sex**			
Female	1.088	0.970	1.220
Male	Reference		
**Race**			
Black	0.791	0.665	0.942
Hispanic	0.841	0.655	1.081
Asian or Pacific Islander	0.748	0.539	1.039
Native American/Other	0.855	0.603	1.214
White	Reference		
**Median household income**			
0-25th percentile	0.639	0.542	0.754
26th to 50th percentile	0.724	0.613	0.855
51st to 75th percentile	0.733	0.620	0.866
76th to 100th percentile	Reference		
**Primary Payer**			
Medicare	0.750	0.643	0.874
Medicaid	0.842	0.684	1.037
Self-Pay/No Charge	1.201	0.899	1.602
Other	2.301	1.735	3.050
Private insurance	Reference		
**Number of Comorbidities**	0.950	0.919	0.983
**Severity of Illness Subclass**			
APR-DRG 0,1, lowest	Reference		
APR-DRG 2	1.159	0.948	1.416
APR-DRG 3	0.988	0.813	1.201
APR-DRG 4, highest	0.686	0.551	0.855
**Surgery**			
No	Reference		
Yes	0.519	0.341	0.791
**Radiation**			
No	Reference		
Yes	1.239	0.958	1.603
**Chemotherapy**			
No	Reference		
Yes	0.713	0.563	0.903

### Association of palliative care with discounted hospital charges and LOS

Table 4 shows the palliative care associations with discounted total hospital charges and LOS. After controlling for other variables, receiving palliative care during hospitalization was associated with statistically significant decreased LOS (β = -0.112, *p* < 0.001), which means 11.2% lower LOS than the not receiving group. Additionally, the use of palliative care was significantly associated with decreased hospital charges (β = -0.284, *p* < 0.001), which means 28.4% lower discounted total costs than the not receiving group after controlling for other variables.

**Table 4:** Results of Survey Regression: Association between palliative care and discounted hospital charges, length of stay

	**Discounted Total Charges**			**Length of Stay**		
	**EST**	**SE**	***P***	**EST**	**SE**	***P***
**Palliative**						
No	Reference			Reference		
Yes	(0.284)	0.027	< .0001	(0.112)	0.025	< .0001
**Age Group**						
40	Reference			Reference		
40–49	(0.078)	0.114	0.492	(0.062)	0.100	0.536
50–59	(0.191)	0.106	0.072	(0.127)	0.092	0.165
60–69	(0.313)	0.106	0.003	(0.166)	0.092	0.070
≥ 70	(0.495)	0.108	< .0001	(0.162)	0.093	0.083
**Sex**						
Female	0.080	0.026	0.003	0.035	0.024	0.152
Male	Reference			Reference		
**Race**						
Black	0.154	0.040	< .0001	0.149	0.037	< .0001
Hispanic	0.304	0.060	< .0001	0.170	0.056	0.003
Asian or Pacific Islander	0.390	0.070	< .0001	0.166	0.072	0.022
Native American/Other	0.131	0.090	0.145	0.223	0.083	0.007
White	Reference			Reference		
**Median household income**						
0-25th percentile	(0.284)	0.038	< .0001	(0.017)	0.036	0.640
26th to 50th percentile	(0.221)	0.039	< .0001	0.019	0.036	0.603
51st to 75th percentile	(0.054)	0.038	0.162	0.011	0.036	0.755
76th to 100th percentile	Reference			Reference		
**Primary Payer**						
Medicare	0.353	0.038	< .0001	0.110	0.034	0.001
Medicaid	0.292	0.047	< .0001	0.155	0.046	0.001
Self-Pay/No Charge	0.150	0.072	0.038	0.158	0.061	0.010
Other	(0.544)	0.069	< .0001	(0.218)	0.059	0.000
Private insurance	Reference			Reference		
**Number of Comorbidities**	0.069	0.008	< .0001	0.062	0.007	< .0001
**Severity of Illness Subclass**						
APR-DRG 0,1, lowest	Reference			Reference		
APR-DRG 2	(0.377)	0.050	< .0001	(0.177)	0.044	< .0001
APR-DRG 3	(0.305)	0.046	< .0001	(0.193)	0.042	< .0001
APR-DRG 4, highest	0.027	0.050	0.589	0.048	0.048	0.313
**Surgery**						
No	Reference			Reference		
Yes	0.744	0.094	< .0001	0.877	0.082	< .0001
**Radiation**						
No	Reference			Reference		
Yes	0.294	0.052	< .0001	0.687	0.044	< .0001
**Chemotherapy**						
No	Reference			Reference		
Yes	0.354	0.049	< .0001	0.779	0.037	< .0001

## Discussion

Through a large-scale national inpatient sample dataset, this study found an increase in palliative care services and a clear association between reduced healthcare utilization and deceased metastatic lung cancer patients who received palliative care. Furthermore, we observed different patient characteristics of those receiving palliative care in the study sample.

Our study results indicate that palliative care use grew during the study periods, which aligns with other studies investigating palliative care utilization among various chronic diseases. For instance, a study reported an increase of 0.45% to 2.56% between 2006 and 2012 among end-stage chronic obstructive pulmonary disease patients [22]. Another study using the National Inpatient Sample reported that chronic obstructive pulmonary disease patients receiving palliative care increased from 8.5% to 57.2% between 2005 and 2014 [23]. In studies using cancer patients, similar trends were also reported. In 'Dev's study, outpatient consultations tripled, and inpatient consultations increased 25 fold from 2000 to 2010 [24]. A recent study investigated palliative care use in metastatic non-small cell lung cancer patients using the Surveillance, Epidemiology, and End Results-Medicare linked database and reported that between 2001 to 2013, the temporal trend of palliative care use drastically increased from 3.6% to 31.9% in the study population [15]. Comparatively, our study revealed that almost 50% of lung cancer patients received palliative care. In the United States, the number of palliative care facilities has significantly grown with pediatric palliative programs and more than 1000 new palliative programs during the past decades [25, 26]. The escalation of cancer and other chronic disease incidences and an increase in life expectancy, promises that the need for palliative care services will continue to grow in the United States and worldwide.

Our study also found new insights on who receives palliative care and how it is associated with efficient healthcare spending and utilization. A recent study also reported that income was significantly associated with palliative care- especially in the top quartile compared to the bottom quartile using NIS data [27]. Economic factors may play an essential role in receiving palliative care, so more research should be conducted on this topic. Health policymakers must learn to promote access to the service which may reduce unnecessary interventions at 'patients' end of life. Other studies also suggested palliative care reduces healthcare cost for seriously ill lung cancer patients [15, 28–30]. However, the datasets of those studies were limited to certain population groups. One strength of our study was using NIS data which contains all age groups, races, and many other socio-economic statuses, representing a broader population; hence this study’s results may be generalized to all patients with lung cancer in the United States.

Although this study has several insights and strengths, there are some limitations worth noting. First, we used a national inpatient dataset which, depending on ICD-9-CM codes, may have a limited capture of palliative care. It is possible that terminal patients were coded with palliative care and were discharged to hospice, thus receiving less interventions. Second, the dataset does not have detailed clinical information such as stage or pharmacologic treatments. However, this study contained control variables, including APR-DRG, surgery, radiation, and chemotherapy, which may play a proxy role in 'patients' status. Third, the dataset of this study may not fully capture whether they are receiving care in an outpatient or inpatient setting and 'patients' or 'physicians' preferences toward palliative care. Further study should be conducted on how this may affect palliative care delivery and its association with spending and utilization. Finally, we were unable to capture the timing of palliative care delivery to patients due to a lack of information in the dataset. This could be another critical issue in seriously ill lung cancer patients. However, given that we conducted research with a well-sampled dataset with multiple study periods, we believe that the findings in our study are generalizable to most US deceased lung cancer patients and promote the benefits of palliative care.

## Conclusion

Clinical evidence has demonstrated the benefits of palliative care. Palliative care is associated with efficient end-of-life healthcare utilization. Health policymakers should be aware of the characteristics of receiving the care and the importance of limited healthcare resource allocation as palliative care will continue to grow in cancer treatment.

## Data Availability

All data generated or analysed during this study are included in this published article.
